# Prevention of commercial tobacco use among Native American youth

**DOI:** 10.3389/fpubh.2026.1823429

**Published:** 2026-07-17

**Authors:** Rose Wimbish-Tompkins, John Lowe, Huigang Liang, Melessa Kelley

**Affiliations:** 1 College of Nursing and Health Innovations, The University of Texas at Arlington, Arlington, TX, United States; 2 School of Nursing, The University of Texas at Austin, Austin, TX, United States; 3 Forman College, The University of Memphis, Memphis, TN, United States

**Keywords:** commercial tobacco use, community-based participatory research, native American youth, native reliance, talking circle intervention

## Abstract

**Objectives:**

Culturally grounded approaches to commercial tobacco use (CTU) prevention among Native American youth remain limited. This pilot study evaluated the feasibility and acceptability of the Talking Circle Intervention (TCI) and explored preliminary trends in CTU and Native Reliance (cultural identity).

**Method:**

A one-group pretest-posttest design was utilized with a community-based participatory research approach. A total of 20 Native American youth ages 10–12 were recruited using nonrandom convenience and snowball sampling approaches from a rural Midwestern tribal community. Primary indicators of feasibility included *a priori* set benchmarks of ≥70% recruitment, ≥ 80% retention, and ≥ 90% data completeness and achievement of criterion (target enrollment, follow-up completion, complete data sets). Exploratory outcomes included CTU and Native Reliance (cultural identity), assessed at baseline and 3-months. Data were analyzed with descriptive statistics, confidence intervals, and single group paired t-test.

**Results:**

Primary indicators of feasibility in terms of predefined criterion were met. Benchmark findings included 100% recruitment within the planned timeframe, 100% retention at 3 months, and 100% data completeness across measures. Exploratory analyses indicated a modest decrease in study participant reported CTU scores and an increase in Native Reliance (cultural identity) scores over the study timeframe.

**Conclusion:**

Although observed trends in CTU and cultural identity suggest potential promise, these results are preliminary and must be interpreted with caution. Further research using a larger sample size and a randomized controlled design to evaluate the effectiveness of the TCI is warranted and will further clarify how cultural identity functions as a protective factor against CTU.

## Introduction

In the United States, commercial tobacco use (CTU) remains a leading cause of preventable morbidity and mortality with Native American youth being particularly vulnerable to early initiation. CTU often leads to long-term addiction and serious health consequences, thereby contributing to significant health disparities among Native American people, including an increased risk of cancer, cardiovascular disease, and respiratory illness (1–6). Native American youth continue to experience disproportionately higher rates of commercial tobacco product use (smokable, smokeless), typically occurring before the age of 12 (1, 7–10). When compared with other racial and ethnic groups, commercial tobacco cessation rates are relatively low among Native American Youth (11–15). Native American high school and middle school students have greater e-cigarette smoking rates than any other racial/ethnic high school and middle school student groups. Approximately 11.5% of Native American high school students report e-cigarette smoking in comparison to 2.3% of Asian, 5.9% of Caucasian, 6.1% of Hispanic/Latino, 6.6% of multiracial, and 7.0% of Black/African American high school students (14). Findings from a study indicated that approximately 16.3% of the Native American high school and middle school students reported the use of commercial tobacco products, compared to 10.0% of Black/African American, 9.0% of multiracial, 8.4% of Hispanic/Latino, 7.8% of Caucasian, and 3.3% of Asian high school and middle school students (14).

Unique health challenges shaped by the combined influences of historical, cultural, social, and environmental factors are faced by many Native American youth. These challenges are compounded by limited access to health care and community resources. The lingering effect of colonization, forced relocation, and cultural suppression have produced intergenerational trauma that continues to affect Native American communities (16–18). This trauma often manifests as elevated rates of depression, anxiety, and stress, which may contribute to higher rates of CTU among Native American youth (16, 18–20). For many of these youth, CTU becomes a coping mechanism for stress, trauma, and isolation, perpetuating cycles of addiction and worsening physical and emotional health outcomes. Many Native American youths experience cultural tension due to the lack of congruency between their traditional Native American values, beliefs, and practices and the dominant sociocultural norms of American society (12, 21). This cultural tension can influence health-related behaviors, identity confusion, and low self-esteem among Native American Youth, potentially contributing to their increasing vulnerability to risk behaviors such as CTU.

Native American youth who live in non-Native American communities may also face racism, discrimination, and bullying, which further heighten emotional distress and may increase the likelihood of CTU as a coping strategy. Confusion between tobacco use for traditional/cultural purposes and the use of harmful commercial tobacco products also contributes to this problem among Native American youth (22, 23). Although there are health risks associated with commercial tobacco use research remains limited regarding its effects among Native American youth populations and the protective role that traditional cultural practices may play in CTU prevention.

Nurses and other health professionals have a responsibility to approach prevention and health promotion using culturally grounded methods that respect and reflect Native American values, beliefs, and practices. Community-based programs are critical for building resilience, fostering cultural pride, and enhancing health awareness from within Native American worldviews. Several interventions designed for Native American youth have either adapted evidence-based approaches or developed novel strategies that are culturally grounded (24–27). While these interventions often demonstrate creativity in using community structures such as schools, emergency departments, and online networks, their effectiveness in improving overall health outcomes has been mixed. Many incorporate cultural elements superficially rather than as foundational principles, which limits their depth of engagement and sustainability (28, 29). To address these gaps, an evidence-based and culturally strength-based group intervention, the Talking Circle Intervention, was developed and piloted over 10 weeks in two schools within a rural Midwestern tribal community. The intervention was designed for Native American youth ages 10–12 and aimed at promoting health awareness and preventing CTU.

Research indicates that maintaining cultural traditions and community connections is linked to reduced health risks among Native American populations (16, 28, 30, 31). Native American cultural beliefs and values play a critical role in shaping tobacco-related behaviors among Native American youth. Traditional tobacco holds sacred and ceremonial importance, but commercial tobacco represents a harmful and non-traditional use. The distinction between these forms of tobacco use in conjunction with historical trauma, social stressors, and cultural disconnection contribute to increased susceptibility to commercial tobacco use (CTU), particularly among Native American youth (5, 20, 29, 32, 33). Strengthening cultural identity, community connection, and traditional knowledge systems may therefore serve as protective factors that reduce CTU risk behaviors and promote healthier decision-making for Native American Youth. However, there are limited studies that have examined how traditional cultural teachings can directly enhance prevention efforts for Native American youth (9, 22, 32). Given the limited evidence on culturally grounded interventions targeting commercial tobacco use among Native American youth, a pilot feasibility study is necessary to assess the implementation processes, acceptability, and exploratory outcomes of effectiveness prior to a fully powered randomized control trial.

## Methods

The primary aim of this pilot study was to evaluate the feasibility of the Talking Circle Intervention (TCI) for Native American youth utilizing primary indicators that included recruitment, retention, and data completeness. A secondary, exploratory aim was used to assess the exploratory (preliminary) outcomes of commercial tobacco use (CTU) and Native Reliance (cultural identity) at baseline and 3-months. Consistent with recommendations for pilot studies, formal hypothesis testing was not the primary objective. However, it was anticipated that the study participants would demonstrate preliminary directional improvements including reductions in CTU-related behaviors and increases in Native Reliance (cultural identity) scores following participation in the TCI.

### Theoretical framework

**Figure 1:**
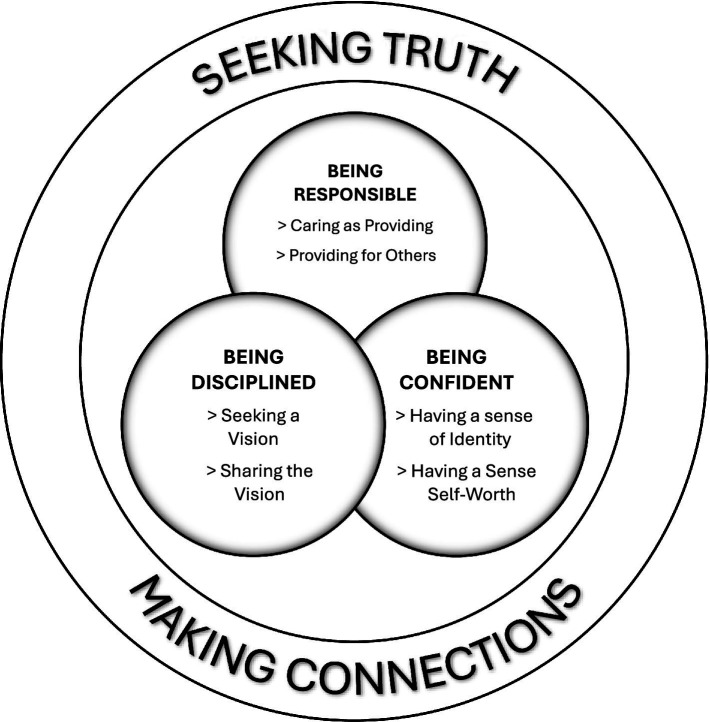
Native reliance theory and guiding framework.

This feasibility pilot study was conceptually guided by Native Reliance, which formed the theoretical basis for the research. Native Reliance is a cultural identity theoretical framework, reflecting the holistic worldview, values, beliefs, and behaviors within Native American culture. Native American leaders and cultural knowledge keepers describe Native Reliance as a holistic way of being that shapes cultural identity, motivates positive behavior choices, and a process that supports health and wellbeing among Native American people (34). The Native Reliance Theory and guiding framework has five key values and beliefs that are illustrated as a circular model. As seen in Figure 1 the model is depicted as: two overarching domains (seeking truth and making connections) and three interrelated concepts (being responsible, being disciplined, and being confident), reflecting the core values and goals of a Native American cultural identity.

### Talking circle intervention

In Native American perspective, the circle is an underlying, foundational concept, and a sacred symbol of the interdependence for all forms of life. Talking Circles are an ancient spiritual practice where the people come together in the sacred circle to connect, tell stories, pray, and heal (35, 36). The Talking Circle Intervention is a means to leverage this traditional cultural practice to help Native American youth move away from harmful ways of coping, toward positive ways of being. The Talking Circle Intervention was delivered to the study participants by a trained facilitator as a 10-session manualized school-based program with one weekly session for 10 weeks. Its content addressed factors associated with commercial tobacco use (CTU) such as stress, anxiety, peer pressure, trauma, and depression, from a cultural strength-based perspective (37–39).

### Study design

This pilot study utilized a one-group pretest-posttest design with a community-based participatory research approach (CBPR). The research team used the CBPR approach of Participatory Action Research (PAR) and its three principles (*Look, Think, Act*) to partner with a Midwestern Tribe and form a Community Partnership Committee (CPC) with eight rural Midwestern Tribal members (40). In the “look phase,” the team engaged with the community to clearly articulate the problem as it exists within the community context. This set the foundation for the “think phase,” where the collaborative partner and assembled CPC views were synthesized by the research team to create an inclusive perspective of the problem to be addressed. Followed by the “act phase” where tasks involving the study process are identified, collaborative partners are engaged in affecting change, and the Talking Circle Intervention for the prevention of commercial tobacco use (CTU) will be implemented and assessed.

### Sample, recruitment, and data collection

Nonrandom convenience and snowball sampling approaches were utilized for the recruitment of 20 Native American youth participants ages 10–12, who were from two rural elementary schools within the boundaries of the participating Midwestern tribal community. The study engaged youth between these ages, because this developmental stage is considered critical for establishing healthy behaviors and cultural identity formation. Additionally, a sample size of 20 participants aligns with methodological recommendations for behavioral pilot and feasibility studies, which support the utilization of small samples to evaluate indicators such as recruitment, retention, intervention adherence, and completeness of assessment measures (41, 42). Based on these recommendations, the sample size was appropriate for producing meaningful feasibility metrics and identifying potential procedural and logistical challenges. Overall, this approach is consistent with best practices and reporting expectations for feasibility research and provides a necessary foundation for the development of more rigorous, adequately powered randomized controlled trials.

### Ethics and approval statements

Approval for this study was obtained from the partnering university’s Institutional Review Board and the collaborating Tribe’s governing body if appropriate. All study procedures followed ethical principles for research involving Native American communities, including respect for tribal sovereignty and cultural protocols. Prior to participation in the study, written informed assent was obtained from all Native American youth participants, along with written informed consent from their parents or legal guardians. Each study participant was assigned a unique identification number that was linked to all collected data and maintained within the study database. To ensure confidentiality, all other databases contained no identifying information.

### Study inclusion and exclusion criteria

Inclusion criteria for participants of the study included those with or without a history of CTU who are: 1. ages 10–12 years, 2. enrolled in the 5th or 6th grade from the participating rural midwestern school, 3. a member of the partnering rural Midwestern Native American Tribe and resident within its community, and 4. able to read, write and speak English. Participants were excluded from the study if they had previously taken part in, or were currently participating in any type of intervention program for the prevention or treatment of commercial tobacco use (CTU).

### Measures

Assessment tools for this pilot study included the Roster and Participation Scale (RPS), Native-Reliance Questionnaire (NRQ), and Global Youth Tobacco Survey (GYTS). The RPS is a 15-item tool used to measure feasibility indicators that include recruitment, retention, and data completeness. NRQ is a 24-item measure to assess cultural identity. The NRQ utilizes a 5-point Likert scale from strongly agree to strongly disagree with a minimum score of 24 and a maximum score of 120 (38). Higher scores reflect a better sense of Native Reliance (cultural identity). The NRQ has a Cronbach alpha of 0.90 and principal component analysis with a Varimax rotation validated for the 3 core concepts of: being responsible, disciplined, and confident (38). NRQ items had high factor loadings on the assigned dimensions (> 0.60) and low factor loadings on other dimensions, confirming discriminant and convergent validity (38). The GYTS has an internal consistency Cronbach alpha of 0.70 to 0.90 (43). Commercial Tobacco Use (CTU) was assessed using selected items adapted from the GYTS. These items included measures of lifetime tobacco use (e.g., ever tried cigarettes or other tobacco products), recent use (e.g., past 30-day use), access and availability, and susceptibility or intention to use. Responses to selected items were coded and combined into a composite CTU index score, with higher values indicating greater tobacco-related risk or engagement. This composite approach is consistent with prior research using GYTS derived comes to capture multidimensional tobacco-related behaviors in youth populations (World Health Organization [WHO], 43). Due to the pilot nature of the study and small sample size, GYTS CTU scores are interpreted as relative outcomes of behavioral patterns rather than precise prevalence estimates.

### Data analysis

The IBM Statistical Package for the Social Sciences (SPSS) was used to perform descriptive statistics (means, standard deviations and confidence intervals) that were calculated to summarize participant characteristics and outcome variables at baseline and 3-month follow-up. To evaluate the preliminary effect of the intervention on Commercial Tobacco Use (CTU) and Native Reliance (cultural identity), paired-samples t-tests were conducted to compare pre-intervention (baseline) and post-intervention (3-month) scores. In consideration of the feasibility focus for this study, its analyses emphasized estimation rather than hypothesis testing. Descriptive statistics and confidence intervals were used to examine preliminary patterns of change over time. Paired-sample t-tests were conducted to explore within-subject change. However, these analyses are interpreted as exploratory and preliminary, not confirmatory. Observed changes are reported as outcomes of potential promise to inform future, adequately powered trials. This design enabled the assessment of preliminary changes over a 3-month time period while prioritizing key feasibility outcomes, including intervention recruitment, retention, engagement, data completeness, and feasibility.

The feasibility of the Talking Circle Intervention for commercial tobacco use prevention for youth participants was examined using outcomes consistent with recommendations for pilot feasibility studies. These outcomes included preliminary intervention effects, assessment completion, and data quality (41, 42). Changes in outcomes over time were examined to assess whether the intervention demonstrated early indications that were beneficial. Commercial tobacco use behaviors were assessed using selected items adapted from the GYTS. Cultural identity and connectedness were assessed using the NRQ. These feasibility outcomes provided insight into both the practicality of implementing the Talking Circle Intervention within a tribal youth setting and the potential for the intervention to promote reduced commercial tobacco use enhanced Native Reliance (cultural identity). Positive trends in these outcome measures are considered evidence of preliminary promise that warrant future testing as a larger scale study.

### Fidelity

Study intervention fidelity was maintained with a Talking Circle Intervention (TCI) session manual, trained facilitator, and TCI Fidelity Monitoring Tool to observe for participant challenges and issues.

## Results

The study successfully recruited the target sample of 20 Native American youth within the planned 3-month period with 25 screened and 20 determined eligible. Retention was 100% of enrolled participants completing both baseline and 3-month follow-up assessments and no withdrawals or loss to follow-up. Study participant recruitment reached 100% of the target sample, exceeding the predefined ≥ 70% benchmark. While study participant retention was 100% and surpassed the ≥ 80% benchmark. Study participant data completeness was 100%, which exceeded the ≥ 90% benchmark. As seen in Table 1, all *a priori* feasibility indicators of recruitment, retention, and data completeness in terms of predefined benchmarks and criterion were met. Overall, the pilot feasibility study findings are promising and support the progression to a full scale trial.

**Table 1:** Talking circle intervention primary indicators of feasibility.

Feasibility domain	Metric	Planned (target)	Observed	Rationale
Recruitment	Target sample size (n)	20	20 (100%)	Target achieved within planned window
	Screened (n)	25	25	Assessed for eligibility during recruitment
	Eligible (n/%)	25 (100%)	20 (80%)	5 did not meet inclusion criteria
	Invited (n)	20	20	Recruitment community flyer distribution
	Consented/Enrolled (n/%)	20 (100%)	20 (100%)	Consent rate = 100% of invited
	Recruitment rate	≥ 70% of target in 3 months	100% in 3 months	Recruitment completed as planned
Retention	Completed baseline (n/%)	100%	20 (100%)	Indication of participant accessibility and willingness to participate
	Completed 3-month follow-up (n/%)	≥ 80%	20 (100%)	Indication of participant acceptability, burden, and study fit
	Lost to follow-up/withdrawals	0	0	No withdrawals/losses to follow-up
Data completeness	Key outcome measures complete (%)	≥ 90%	100%	No missing survey data
	Overall dataset completeness (%)	≥ 90%	100%	All participants completed required measures

Feasibility decision	Benchmark/Predefined percentage	Percentage met	Criterion	Criterion met
	Recruitment = ≥ 70%	Yes	Target enrollment achieved	Yes
	Retention = ≥ 80% at final follow-up	Yes	Full follow up completed	Yes
	Data completeness = ≥ 90%	Yes	Fully completed data sets	Yes

The study findings also revealed that the Talking Circle Intervention (TCI) demonstrated preliminary promise in decreasing commercial tobacco use among participants. At baseline, the average commercial tobacco use (CTU) score was 5.25, with considerable variability (standard deviation = 6.37). At 3 months, mean CTU scores decreased slightly to 4.6, and the variability decreased as well (standard deviation = 5.56). The difference between the baseline and 3-month values is 0.65, which suggests a slight reduction in CTU. The confidence interval for this difference (0.12 to 1.18) does not include 0, indicating that the observed reduction in CTU may potentially reflect a meaningful change. The paired-sample t-test indicated a modest decrease in CTU scores from baseline to 3 months (mean difference = 0.65, 95% CI [0.12, 1.18], t (19) = 2.56, *p* = 0.019). Overall, the observed reduction in CTU scores reflected modest decreases in self-reported tobacco-related behaviors and/or susceptibility, instead of a definitive reduction in tobacco use occurrence (See Table 2).

**Table 2:** Marginal means: changes in commercial tobacco use.

Variable	Obs.	Mean	Std. Err.	Std. Dev.	95% Conf. interval (CI)	
CTU					CI lower	CI upper
Time						
baseline	20	5.25	1.425104	6.37223	2.267223	8.232777
3-months	20	4.6	1.242663	5.557356	1.999077	7.200923
diff	20	0.65	0.2541757	1.136708	0.1180042	1.181996

*p* = 0.019.

The TCI results reveal that at baseline, Native Reliance (cultural identity) has an average score of 96.3, with a moderate amount of variability (standard deviation = 11.25). At 3 months, Native Reliance (cultural identity) increased to an average score of 106.4, with a slightly smaller amount of variability (standard deviation = 9.93). The difference in Native Reliance (cultural identity) between baseline and 3 months is −10.1, which represents an increase in cultural identity over the 3-month period. The confidence interval for the change (−13.69 to −6.51) does not include 0, suggesting that the observed increase may represent a meaningful change and support the preliminary promise of the intervention. The paired-samples t-test indicated an increase in Native Reliance (cultural identity) scores from baseline to 3 months (mean difference = −10.1, 95% CI [−13.69, −6.51], t(19) = −5.89, *p* < 0.0001). Overall, the results indicate a positive change in Native Reliance (cultural identity) among participants from baseline to 3 months (See Table 3).

**Table 3:** Marginal means: changes of native reliance (cultural identity).

Variable	Obs.	Mean	Std. Err.	Std. Dev.	95% Conf. interval (CI)	
Native Reliance (cultural identity)					CI lower	CI upper
Time						
baseline	20	96.3	2.515321	11.2488	91.03537	101.5646
3-months	20	106.4	2.22119	9.933463	101.751	111.049
diff	20	−10.1	1.713568	7.66331	−13.68654	−6.513461

*p* < 0.0001.

## Discussion

The primary aim of this pilot study was to evaluate the feasibility and acceptability of implementing the Talking Circle Intervention (TCI) among Native American youth. Findings indicated that all predefined benchmarks and criterion in terms of primary feasibility indicators (recruitment, retention, data completeness) were met. In addition to the primary indicators of feasibility, exploratory analyses examined trends in commercial tobacco use (CTU) and Native Reliance (cultural identity). Observed patterns indicated a modest decrease in self-reported CTU scores and an increase in Native Reliance (cultural identity) over the 3-month period. While these findings are encouraging, these findings must be interpreted as preliminary trends rather than evidence of effectiveness due to the small sample size, lack of a control group, and pilot study design.

The observed increase in Native Reliance is consistent with previous research conducted that has indicated how cultural identity, connectedness, and engagement in traditional practices may serve as protective factors for Native American youth (34, 38, 39). The TCI and its process are grounded in storytelling, shared dialogue, and cultural teachings provide a supportive space for youth to reflect on health behaviors and choices within the context of their lives and cultural beliefs and values. Although this process was theoretically aligned with Native Reliance (cultural identity), the study method was not intended as a means to test intermediate processes or causal relationships. The observed decrease in CTU-related scores may reflect increased awareness, shifts in attitudes, or changes in susceptibility rather than decisive behavioral change. Given that CTU was assessed using a composite index of participant reported behaviors and intentions, these findings should be interpreted with caution and seen as indicators of potential promise that warrant further investigation.

### Limitations

These findings should be considered within the context of several limitations. First, the small sample size (*N* = 20) and use of a single Native American tribal community limit generalizability. Second, the nonrandomized one-group design without a control group prohibits causal inference and limits the ability to attribute observed changes to the intervention Third, all measures relied on self-reporting, which may introduce recall and social desirability bias, particularly within a group-based cultural intervention context. Fourth, the brief follow-up period (3-months) limits the understanding of the Talking Circle Intervention (TCI) with respect to its long-term sustainability. Fifth, dependance on self-reported measures may introduce recall and social desirability bias, particularly within a culturally grounded group intervention context such as Talking Circles, where participants may feel motivated to provide socially acceptable responses. Finally, the study was not powered to examine subgroup differences (e.g., by site or baseline tobacco use status). Finally, CTU behaviors and cultural identity development are complex and dynamic processes that evolve over time and may be influenced by broader social, cultural, and environmental factors not captured in the present study.

### Implications for future research

Future research should explore the long-term sustainability of the Talking Circle Intervention (TCI) and examine the mechanisms through which cultural identity exerts its protective influence on commercial tobacco use (CTU) prevention. Future studies should include larger, more diverse samples, longer follow-up periods, and randomized controlled designs to evaluate intervention effectiveness. Incorporating mixed-method approaches, including qualitative inquiry, would provide deeper insight into participant experiences and mechanisms of change in relation to CTU prevention. Additionally, future research should include more detailed behavioral outcome measures, including frequency distributions and subgroup analyses, to better characterize the TCI impact.

## Conclusion

The findings from this pilot study highlight the promise of culturally grounded interventions such as the Talking Circle in supporting positive health behaviors and cultural identity among Native American youth. High levels of recruitment, retention, and data completeness indicate that the study design and process were well-aligned with the Native American community context and participant needs. Exploratory findings demonstrated observed trends toward decreased participant reported commercial tobacco use and increased Native Reliance (cultural identity). These results should be interpreted cautiously given the small sample size, single-group design, and short observation period in which no causal inferences regarding intervention effectiveness can be made.

This study contributes to a growing body of evidence stressing the importance of culturally responsive and community-engaged interventions for Native American youth. The integration of traditional practices, such as Talking Circles, within prevention efforts may offer a meaningful pathway for supporting wellbeing, and positive health behaviors and choices. Future research can build on these findings by including a longitudinal randomized controlled design with a larger more diversified sample to rigorously evaluate the effectiveness, sustainability, and practicality for using the Talking Circle Intervention to prevent CTU among Native American youth. This approach will also further clarify how cultural identity functions as a protective factor against CTU.

## Data Availability

The datasets presented in this article are not readily available because tribal and Indigenous communities maintain governance over data sharing and access and maintain governance and decision making on access of the study data set. Requests to access the datasets should be directed to john.lowe@austin.utexas.edu.
